# Left ventricle restoration in end stage ischemic dilated cardiomyopathy

**DOI:** 10.1186/1749-8090-8-S1-O147

**Published:** 2013-09-11

**Authors:** Z Jonjev

**Affiliations:** 1 Institute for CVD of Vojvodina, Sremska Kamenica, Serbia

## Background

Patients with end stage dilated cardiomyopathy exhibit extensive remodeling of the left ventricle, annular dilation, and significant mitral and tricuspid regurgitation. These changes increase perioperative morbidity and mortality, and emphasize patient candidacy for heart transplantation. We previously published that Reductive Annuloplasty of Double (mitral and tricuspid) Orifices could be successfully applied in selective patients. However, it was unclear if implantation of the artificial mitral valve with preservation of native mitral valve could be used as a method in such cases.

## Methods

There were 16 patients (13 males, 3 females) with ischemic dilated cardiomyopathy, mean ejection fraction below 30% (26.6±3.1%), and mean left ventricle end-diastolic internal diameter greater than 7.0cm (7.3±0.3 cm) with coaptation depth of the mitral valve (tenting area) significantly lower than 1.1cm. In addition to myocardial revascularization mechanical or biological artificial valve were implanted in 12 and 4 patients respectively. Modified De Vega tricuspid annuloplasty was performed in all patients.

## Results

There was no postoperative 30-day mortality. Survival rate after 5 years was 93.75%.

## Conclusion

Reverse remodeling of the left ventricle by CABG surgery followed with implantation of the artificial mitral valve and tricuspid suture annuloplasty could be successfully applied in selective patients with ischemic dilative cardiomyopathy. Our method should not be recognized as a valve repair, but ventricular repair procedure also.

